# Defining the Vulnerable Period for Re-Establishment of *Clostridium difficile* Colonization after Treatment of *C. difficile* Infection with Oral Vancomycin or Metronidazole

**DOI:** 10.1371/journal.pone.0076269

**Published:** 2013-10-02

**Authors:** Turki Abujamel, Jennifer L. Cadnum, Lucy A. Jury, Venkata C. K. Sunkesula, Sirisha Kundrapu, Robin L. Jump, Alain C. Stintzi, Curtis J. Donskey

**Affiliations:** 1 Ottawa Institute of Systems Biology, Department of Biochemistry, Microbiology and Immunology, Faculty of Medicine, University of Ottawa, Ontario, Canada; 2 Research Service, Cleveland Veterans Affairs Medical Center, Cleveland, Ohio, United States of America; 3 Geriatric Research, Education and Clinical Center, Cleveland, Ohio, United States of America; 4 Department of Medicine, Infectious Diseases Division, Case Western Reserve University School of Medicine, Cleveland, Ohio, United States of America; Universidad Andres Bello, Chile

## Abstract

**Background:**

*Clostridium difficile* is an anaerobic, spore-forming bacterium that is the most common cause of healthcare-associated diarrhea in developed countries. A significant proportion of patients receiving oral vancomycin or metronidazole for treatment of Clostridium difficile infection (CDI) develop recurrences. However, the period of vulnerability to re-establishment of colonization by *C. difficile* after therapy is not well defined.

**Principal Findings:**

In a prospective study of CDI patients, we demonstrated that most vancomycin-treated patients maintained inhibitory concentrations of vancomycin in stool for 4 to 5 days after therapy, whereas metronidazole was only detectable during therapy. From the time of elimination of the antibiotics to 14 to 21 days after therapy, a majority of stool suspensions supported growth of *C. difficile* and deep 16S rRNA sequencing demonstrated persistent marked alteration of the indigenous microbiota. By 21 to 28 days after completion of CDI treatment, a majority of stool suspensions inhibited growth of *C. difficile* and there was evidence of some recovery of the microbiota.

**Conclusions:**

These data demonstrate that there is a vulnerable period for re-establishment of *C. difficile* colonization after CDI treatment that begins within a few days after discontinuation of treatment and extends for about 3 weeks in most patients.

## Introduction

*Clostridium difficile* is the most important cause of healthcare-associated diarrhea in developed countries [1]. Oral vancomycin and metronidazole are the agents most often used to treat Clostridium difficile infection (CDI) [1]. These antibiotics are effective in suppressing *C. difficile*, but they are nonselective agents that also cause significant disruption of the indigenous microbiota of the colon [2-4]. For example, oral vancomycin achieves high concentrations in the intestinal tract, resulting in suppression of anaerobic organisms, including *Bacteroides* spp. [4,5]. Such disruption of the indigenous microbiota may predispose patients to recurrent CDI due to regrowth of the original infecting *C. difficile* strains or acquisition of new strains [1]. Although it is known that recurrences may occur days to weeks after completion of CDI therapy, the period of vulnerability to re-establishment of colonization by *C. difficile* after therapy is not well defined. In theory, this period will extend from the time vancomycin or metronidazole levels reach subinhibitory concentrations in the colon to the time when the intestinal microbiota recovers sufficiently to inhibit *C. difficile* growth. A better understanding of the timing of the vulnerable period after CDI therapy is needed for the design of interventions to reduce recurrences. For example, administration of biotherapeutic agents such as nontoxigenic *C. difficile* could be targeted to coincide with the vulnerable period for re-establishment of colonization [6].

Limited information is available regarding the duration of excretion of vancomycin and metronidazole in stool after treatment. Edlund et al. [4] administered oral vancomycin 0.5 gram/day for 7 days to healthy volunteers; the mean concentration of vancomycin in stool after 7 days of treatment was 520 + 197 µg/g, but only 1 of 10 subjects had high levels of vancomycin in stool 1 week after discontinuation of treatment (i.e., 135 µg/g). Metronidazole achieved much lower concentrations in stool of CDI patients during therapy (mean + SD, 9.3 + 7.5 µg/g), and levels became undetectable as diarrhea resolved during treatment [7].

Here, we examined the duration of excretion of vancomycin and metronidazole in stool after treatment of CDI and evaluated the period of vulnerability to re-establishment of colonization by *C. difficile*. Because patients with CDI are often elderly and have received multiple prior courses of broad-spectrum antibiotics, we hypothesized that they may have a prolonged period of vulnerability to *C. difficile* colonization after oral vancomycin or oral metronidazole treatment.

## Materials and Methods

### Ethics Statement

The Institutional Review Board of the Cleveland Veterans Affairs Medical Center approved the study protocol. *C. difficile* strains isolated from patients were collected from the Cleveland VA Medical Center. Written informed consent was obtained from all subjects.

### *C. difficile* Strains

One epidemic North American pulsed-field gel electrophoresis type 1 (NAP1) /polymerase chain reaction ribotype 027 strain (VA 17) and one nonepidemic strain (VA 6) were tested in an in vitro assay of colonization resistance. Both strains were cultured from patients with CDI at the Cleveland VA Medical Center.

### Setting and Study Design

We performed an 8-month prospective, observational study of patients treated for CDI with oral metronidazole or vancomycin at the Cleveland VA Medical Center. Patients receiving therapy for multiple recurrences or for severe, complicated CDI as defined by Cohen et al. [1] (i.e., hypotension or shock, ileus, megacolon) were excluded, but patients with severe but uncomplicated CDI were not. Severe CDI was defined as a case in which the white blood cell count was >15,000 cells/µl and/or the creatinine was increased 1.5 or more times the baseline level [1]. During the study, diagnostic testing for CDI was performed using enzyme immunoassay (EIA) for glutamate dehydrogenase (Wampole C. diff Chek-60, Inverness Medical, Princeton, NJ) as an initial screen with a commercial PCR test for toxin B genes (Becton Dickinson, Cockeysville, MD) for confirmation. The laboratory rejected formed stool samples. The choice of therapy for CDI was made by the physicians caring for the patients. Information regarding the demographic characteristics, coexisting illnesses, and antibiotic therapy was obtained through standardized chart review. The stool sample used for diagnosis of CDI was collected from the clinical microbiology laboratory and additional stool samples were collected every 3-4 days during treatment and for up to 4 weeks after treatment while the patients were hospitalized.

### Microbiology and Molecular Typing

The presence of *C. difficile* in stool samples was measured by plating serially-diluted samples on *C. difficile* Brucella agar (CDBA) as previously described [8]. Plates were incubated for 48 hours at 37 °C in the anaerobic chamber. Colonies were confirmed to be *C. difficile* on the basis of typical odor and appearance of colonies and by a positive reaction using *C. difficile* latex agglutination (Microgen Bioproducts, Camberly, UK). Isolates from the highest dilution with growth were tested for in-vitro cytoxin production using *C. difficile* Tox A/B II (Wampole Laboratories); isolates that did not produce toxin were excluded from the analysis.

Stool isolates were subjected to molecular typing to determine the prevalence of epidemic North American pulsed-field gel electrophoresis type 1 (NAP1) strains. Crude DNA was extracted from *C. difficile* isolates using QIAamp DNA Mini Kit (Qiagen, Valencia, CA) according to the manufacturer’s instructions. Polymerase chain reaction (PCR)-ribotyping was used to genotype *C. difficile* isolates [9]. PCR was performed to amplify one of the genes for binary toxin (*cdtB*) using the methods of Terhes et al [10]. For each assay, a known NAP1 strain was used as a positive control and American Type Culture Collection (ATCC) *C. difficile* 9689 was used as a negative control.

### Measurement of Drug Concentrations in Stool

For vancomycin-treated patients, the concentration of vancomycin in stool samples was measured using an AxSym II fluorescence polarization immunoassay, a standard assay used by clinical Pathology Laboratories for measurement of vancomycin in serum samples. The assay was modified for stool samples by including an extraction of the sample in dilute ammonia to prevent nonspecific binding of vancomycin to protein in the stool [11]. The limit of detection of the assay was 2 µg of vancomycin per mL. For metronidazole-treated patients, the concentration of metronidazole in stool was measured using a bioassay with *Clostridium perfringens* as the indicator organism [12]. For the metronidazole assay, samples from patients receiving concurrent systemic antibiotics for indications other than CDI were excluded. The limit of detection of the assay was 0.5 µg of metronidazole per gm of stool.

### *In vitro* Assay of Colonization Resistance

To assess functional recovery of the ability of the microbiota to inhibit *C. difficile* (i.e., provide colonization resistance) after vancomycin or metronidazole treatment, we used a modification of the *in vitro* assay of colonization resistance developed by Borriello and Barclay [13]. One epidemic NAP1 strain (VA 17) and one nonepidemic strain (VA 6) were tested. Fresh stool samples were homogenized in a 1:1 dilution of pre-reduced sterile water and inoculated with 10^4^ colony-forming units (CFU) of vegetative *C. difficile* inside an anaerobic chamber (Coy Laboratories, Grass Lake, MI). Samples from patients receiving concurrent systemic antibiotics for indications other than CDI and samples with detectable levels of *C. difficile* prior to the inoculation of vegetative *C. difficile* were excluded. The samples were incubated at 37 °C for 24 hours and then serially-diluted and plated on CDBA plates to determine the concentration of *C. difficile*. Loss of colonization resistance was defined as >1 log_10_CFU increase in the *C. difficile* concentration. In addition to the microbiota, residual metronidazole or vancomycin in stool samples could suppress growth of inoculated *C. difficile*; therefore, growth of the *C. difficile* isolates was concurrently assessed in sterile filtrates of the stool suspensions. The filtrates were produced by centrifuging the suspensions at 10,000 rpm for 10 minutes, followed by filtering the supernatant through a 0.22 µm filter.

### Analysis of Stool Microbiota Using Deep 16S rRNA Sequencing

Deep 16SrRNA sequencing was performed on stool specimens from a subset of vancomycin-treated patients with samples available before, during, and after CDI therapy who did not receive concomitant non-CDI antibiotics during or after CDI therapy. DNA was extracted from fecal samples using FastDNA^®^ Spin Kit (MP Biomedicals) utilizing two mechanical lysis cycles in FastPrep^®^ Instrument (MP Biomedicals) at speed 6.0 for 40 seconds. Extracted DNA was then used for library construction. Two sets of primers were designed (Table 1). In set 1, the sequence of V6 universal primers was modified by adding barcodes of 4 to 6 nucleotides, to allow for multiplexing, and the Illumina paired-end sequencing adapters as previously described [14]. In total, 12 different barcodes were generated for each end. Set 2 was designed to have the Illumina paired-end sequencing adapter and the flow-cell adapters.

**Table 1:** Sequence of polymerase chain reaction (PCR) primers used for construction of the Illumina library.

**Primer Set**	**Primer Name**	**Primer Sequence**			
**Set 1**		**Paired-end sequencing adapter**		**Barcode sequences**	**V6 universal primers Adapted from [14]**
	GF1	5’-ACACTCTTTCCCTACACGACGCTCTTCCGATCT		ATAGCG	CAACGCGARGAACCTTACC-3’
	GF2	5’-ACACTCTTTCCCTACACGACGCTCTTCCGATCT		AGGGT	CAACGCGARGAACCTTACC-3’
	GF3	5’-ACACTCTTTCCCTACACGACGCTCTTCCGATCT		TTCAT	CAACGCGARGAACCTTACC-3’
	GF4	5’-ACACTCTTTCCCTACACGACGCTCTTCCGATCT		GATCGT	CAACGCGARGAACCTTACC-3’
	GF5	5’-ACACTCTTTCCCTACACGACGCTCTTCCGATCT		GCCCGT	CAACGCGARGAACCTTACC-3’
	GF6	5’-ACACTCTTTCCCTACACGACGCTCTTCCGATCT		CTGTC	CAACGCGARGAACCTTACC-3’
	GF7	5’-ACACTCTTTCCCTACACGACGCTCTTCCGATCT		CACGT	CAACGCGARGAACCTTACC-3’
	GF8	5’-ACACTCTTTCCCTACACGACGCTCTTCCGATCT		CGTACG	CAACGCGARGAACCTTACC-3’
	GF9	5’-ACACTCTTTCCCTACACGACGCTCTTCCGATCT		GGAC	CAACGCGARGAACCTTACC-3’
	GF10	5’-ACACTCTTTCCCTACACGACGCTCTTCCGATCT		TAGA	CAACGCGARGAACCTTACC-3’
	GF11	5’-ACACTCTTTCCCTACACGACGCTCTTCCGATCT		TCAT	CAACGCGARGAACCTTACC-3’
	GF12	5’-ACACTCTTTCCCTACACGACGCTCTTCCGATCT		ACTT	CAACGCGARGAACCTTACC-3’
	GR1	5’-CTCGGCATTCCTGCTGAACCGCTCTTCCGATCT		ATAGCGA	CAACACGAGCTGACGAC-3’
	GR2	5’-CTCGGCATTCCTGCTGAACCGCTCTTCCGATCT		AGGGTA	CAACACGAGCTGACGAC-3’
	GR3	5’-CTCGGCATTCCTGCTGAACCGCTCTTCCGATCT		TTCATA	CAACACGAGCTGACGAC-3’
	GR4	5’-CTCGGCATTCCTGCTGAACCGCTCTTCCGATCT		GATCGTA	CAACACGAGCTGACGAC-3’
	GR5	5’-CTCGGCATTCCTGCTGAACCGCTCTTCCGATCT		GCCCGTA	CAACACGAGCTGACGAC-3’
	GR6	5’-CTCGGCATTCCTGCTGAACCGCTCTTCCGATCT		CTGTCA	CAACACGAGCTGACGAC-3’
	GR7	5’-CTCGGCATTCCTGCTGAACCGCTCTTCCGATCT		CACGTA	CAACACGAGCTGACGAC-3’
	GR8	5’-CTCGGCATTCCTGCTGAACCGCTCTTCCGATCT		CGTACGA	CAACACGAGCTGACGAC-3’
	GR9	5’-CTCGGCATTCCTGCTGAACCGCTCTTCCGATCT		GGACA	CAACACGAGCTGACGAC-3’
	GR10	5’-CTCGGCATTCCTGCTGAACCGCTCTTCCGATCT		TAGAA	CAACACGAGCTGACGAC-3’
	GR11	5’-CTCGGCATTCCTGCTGAACCGCTCTTCCGATCT		TCATA	CAACACGAGCTGACGAC-3’
	GR12	5’-CTCGGCATTCCTGCTGAACCGCTCTTCCGATCT		ACTTA	CAACACGAGCTGACGAC-3’
**Set2**		**Illumina flow cell adapter**	**Paired-end sequencing adapter**		
	PCRFWD1	5’-AATGATACGGCGACCACCGAGATCT	ACACTCTTTCCCTACACGACGCTCTTCCGATCT-3’		
	PCRRVS1	5’-CAAGCAGAAGACGCCATACGAGATCGGT	CTCGGCATTCCGTCTGAACCGCTCTTCCGATCT -3’		

Set 1 was used in the PCR 1^st^ step and set 2 was used in the 2^nd^ step.

### Illumina Library Construction

The V6 hypervariable region of 16S rRNA was PCR-amplified from extracted DNA in two steps as described by Arthur et al. [14]. Briefly, step 1 used set 1 primers allowing for a unique combination of bar codes for each sample. The PCR reaction contained 50 ng of extracted DNA. PCR reactions were held at 94 °C for 3 min followed by 10 amplification cycles using a touchdown protocol with denaturation at 94 °C for 45 s, annealing at 61 °C for 45 s with 1 °C drop in each cycle, and an extension at 72°C for 45 s. Amplification was continued afterward with additional 15 cycles using 51 °C as annealing temperature. Then, PCR was terminated with a final elongation at 72 °C for 2 min. In the 2^nd^ PCR, 15 µl of the 1^st^ PCR products were utilized with set 2 primers using the same concentrations of PCR reagents. Reaction started with one step at 94 °C for 3 min, with amplification proceeding for 15 cycles at 94 °C for 45 s, 65 °C for 45 s, and 72 °C for 45 s with a final extension step at 72 °C for 2 min. The DNA concentration of cleaned PCR amplicons from the second PCR reaction was quantified, and an equimolar combination of each reaction tube was prepared. Finally, the library was sequenced at The Centre for Applied Genomics at the Hospital for Sick Children in Toronto, Canada using one lane of a HiSeq 2000 to generate paired-end reads of 2x 100 bases. The Illumina reads were deposited in the Sequence Reads Archive at National Center for Biotechnology Information (NCBI) under accession number SRP028823.

### 16S rRNA Sequence Analysis and Characterization of Microbiota Composition and Diversity

Analysis of the sequencing reads was conducted as follows. First, paired-end reads were merged into extended reads using the Fast Length Adjustment of SHort reads algorithm (FLASH) and the fastq file as input [15]. More than 95% of the reads were efficiently merged with a perfect overlap between 10 and 80 nucleotides. Reads that failed to overlap were discarded. Second, reads were extracted and binned by their 5’ and 3’ barcodes using the NovoBarCode command (www.novocraft.com). During this process, the barcode sequences were stripped from the classified reads. Third, the reads were quality filtered using the fastq_quality_filter command from the Fastx toolkit (Version 0.0.13.1; http://hannonlab.cshl.edu/) with a minimum quality score of 33. Finally, the quality filtered classified reads were analyzed using Quantitative Insights Into Microbial Ecology (QIIME) software version 1.5.0 [16]. Specifically, reads were clustered into Operational Taxonomic Units (OTUs) by running a closed-reference OTU picking process using UCLUST against the Greengenes database (version 4feb2011) at 97% sequence identity. The OTUs inherited the taxonomy associated with the corresponding Greengenes sequences. After removal of singletons and doubletons, the relative abundance of taxa (from the phylum to the genus levels) was determined for each sample using the QIIME package. OTUs or taxa exhibiting significant differential representation between groups were selected by linear discriminant analysis using the LEfSe package [17].

### Data Analysis

Data were analyzed using STATA 9.0 (StataCorp, College Station, TX). Continuous data were analyzed using unpaired *t* tests and categorical data were assessed using Fisher’s exact test. Differences in microbiota relative abundance were analyzed using two-tailed Mann-Whitney test.

## Results

### Comparison of Vancomycin and Metronidazole-Treated CDI Patients

During the study period, 76 CDI patients had stool samples available for analysis (range, 1 to 8 stool samples), including 42 (55%) patients treated with oral vancomycin and 34 (45%) patients treated with oral metronidazole. Table 2 shows a comparison of the characteristics of the metronidazole and vancomycin-treated patients. There were no statistically significant differences between the two groups. More than half of *C. difficile* isolates from both treatment groups were NAP1/ribotype 027 isolates based on PCR ribotyping and amplification of the binary toxin gene *cdtB.*

**Table 2:** Patient Characteristics.

Characteristic	Metronidazole N = 34	Vancomycin N = 42	*P-value*
			
Age, years, mean (range)	68 (60- 90)	64 (58–89)	0.17
Male sex	32 (94)	41 (98)	0.58
Creatinine, mg/dL, mean (range)	1.3 (0.3 - 3.9)	1.6 (0.6 - 6.1)	0.33
White blood cell count, cells/mm^3^, mean, (range)	13.5 (1.2–30.9)	12.2 (1.6–33.1)	0.52
On systemic antibiotics during or after treatment	8 (24)	7 (17)	0.57
Diabetes Mellitus	9 (26)	21 (50)	0.06
Coronary Artery Disease	10 (29)	14 (33)	0.81
Chronic Obstructive Pulmonary Disease	6 (18)	7 (17)	1.0
End-Stage Renal Disease	3 (9)	3 (7)	1.0
Severe *Clostridium difficile* Infection	11 (32)	16 (38)	0.64
Infection with epidemic NAP1 strain*	8 (67)	13 (72)	1.0
Nursing home resident	5 (15)	10 (24)	0.39

Notes:Data are No. (%) of patients, unless otherwise indicated.

Abbreviations: NAP1, North American pulsed-field gel electrophoresis type 1.

* Molecular typing was performed on 12 isolates from metronidazole-treated patients and 18 isolates from vancomycin-treated patients.

### Recovery of *C. difficile* From Stool of Vancomycin and Metronidazole-Treated Patients

**Figure 1:**
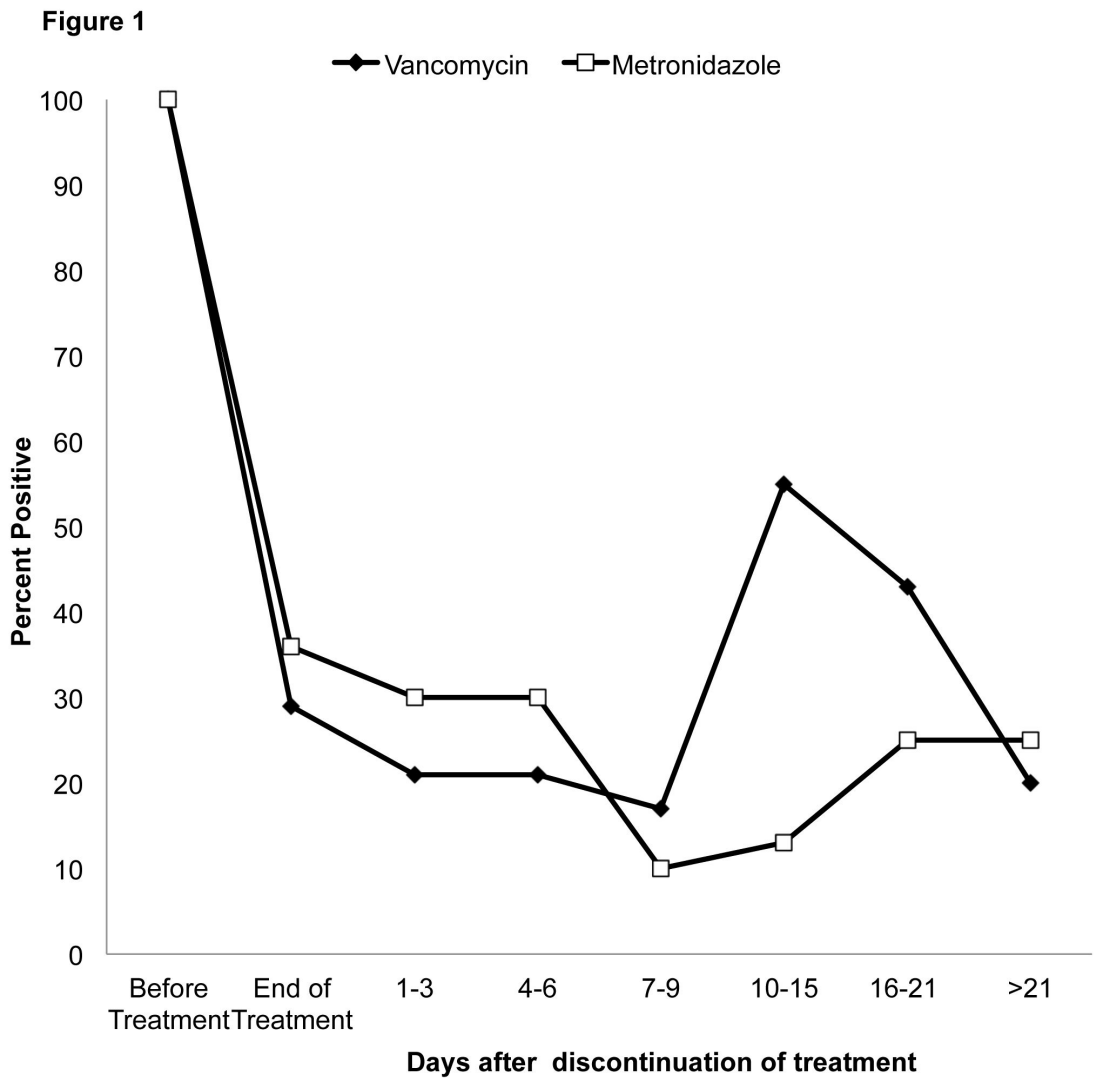
Frequency of recovery of toxigenic *Clostridium difficile* from stool samples of vancomycin and metronidazole-treated patients. In addition to acquisition of toxigenic strains, 2 vancomycin-treated patients acquired colonization with nontoxigenic *C. difficile* between 8 and 14 days after discontinuation of therapy.

Figure 1 shows the frequency of recovery of *C. difficile* from stool samples of vancomycin and metronidazole-treated patients. Vancomycin-treated patients had detectable *C. difficile* in stool less frequently than metronidazole-treated patients at the end of CDI therapy but the difference was not statistically significant (4 of 19, 21% versus 5 of 14, 36%; *P* =0.44); however, vancomycin-treated patients were significantly more likely than metronidazole-treated patients to have detectable *C. difficile* at 10 to 15 days after completion of therapy (6 of 11, 55% versus 2 of 16, 13%; *P* = 0.03). In addition to acquisition of toxigenic strains, 2 vancomycin-treated patients acquired colonization with nontoxigenic *C. difficile* between 8 and 14 days after discontinuation of therapy, whereas none of the metronidazole-treated patients acquired nontoxigenic *C. difficile*.

### Antibiotic Concentrations

**Figure 2:**
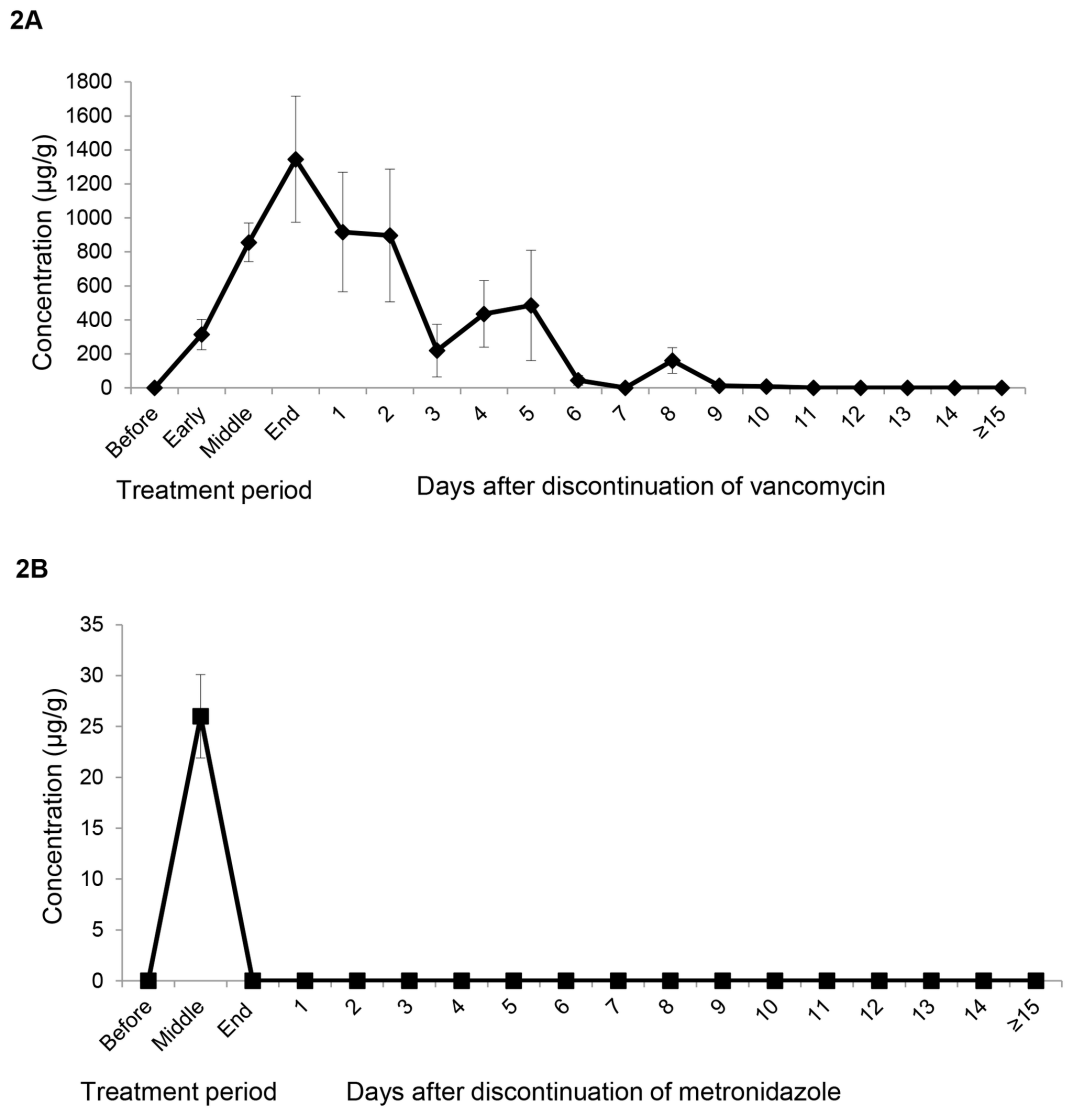
Concentrations of vancomycin and metronidazole in stool during and after Clostridium difficile infection (CDI) treatment. The concentration of vancomycin was measured using an AxSym II fluorescence polarization immunoassay that was modified for stool samples by including an extraction of the sample in dilute ammonia to prevent nonspecific binding of vancomycin to protein in the stool. The limit of detection of the assay was 2 µg of vancomycin per ml. The concentration of metronidazole in stool was measured using a bioassay with *Clostridium perfringens* as the indicator organism. Samples from patients receiving concurrent systemic antibiotics for indications other than CDI were excluded.

Figure 2 shows the concentrations of vancomycin and metronidazole present in stool during and after CDI treatment. High concentrations of vancomycin were present during therapy and detectable levels persisted in stool for up to 8 days after therapy. Metronidazole was detectable in a majority of mid-treatment stool samples (12 of 13 samples tested had detectable metronidazole). However, none of the 26 samples tested at the end of treatment or after treatment had detectable metronidazole.

### *In vitro* Assay of Colonization Resistance

**Figure 3:**
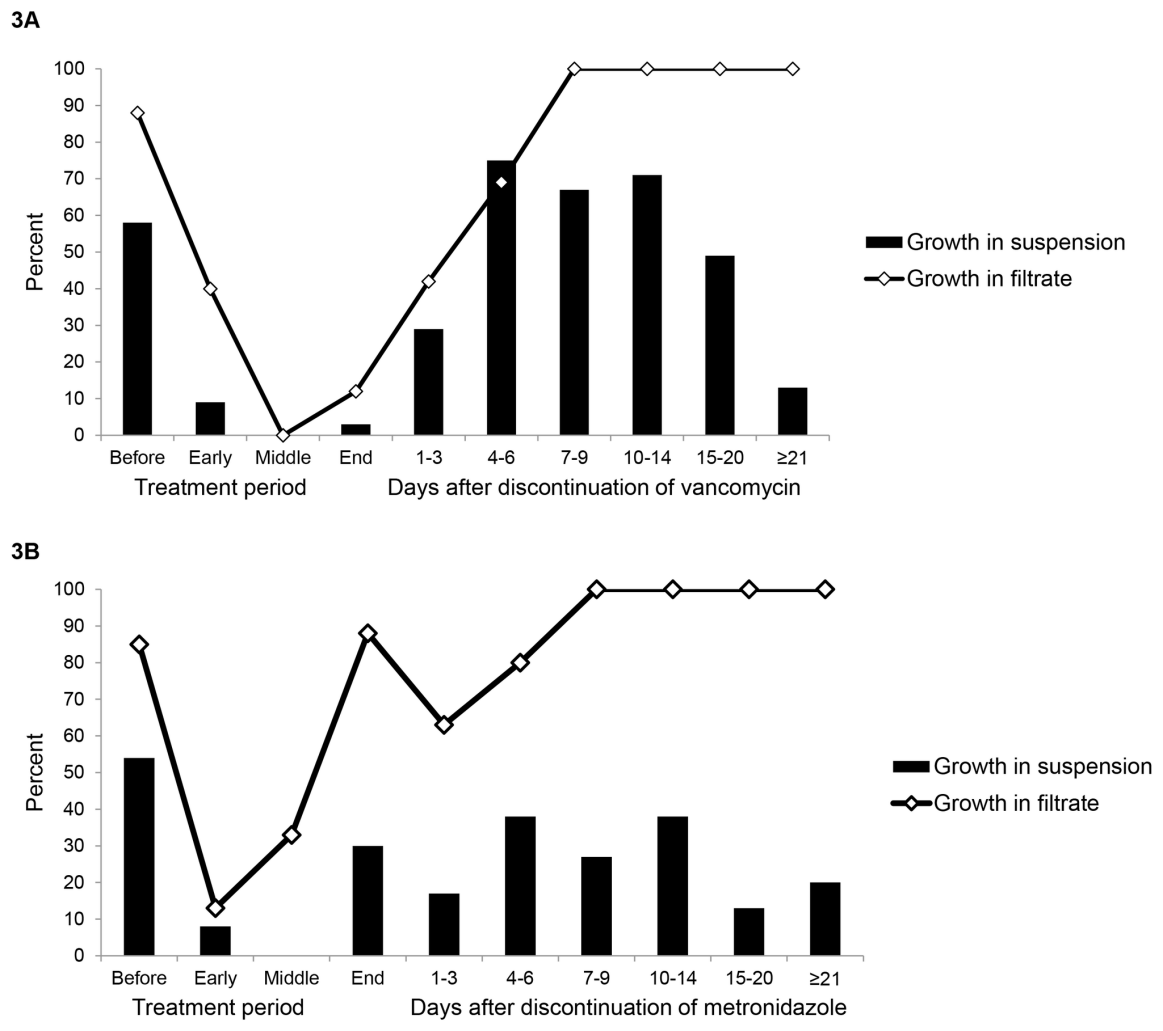
*In vitro* assay of colonization resistance during and after oral vancomycin (A) or metronidazole (B) treatment. Growth of *Clostridium difficile* was assessed in homogenized fresh stool specimens inoculated with 10^4^ colony-forming units (CFU) of vegetative *C. difficile*. Loss of colonization resistance was defined as >1 log_10_CFU increase in the *C. difficile* concentration. To assess whether residual metronidazole or vancomycin in stool samples could account for suppression of growth of inoculated *C. difficile*, growth of the *C. difficile* isolates was concurrently assessed in sterile filtrates of the stool suspensions.

Figure 3 shows results for the *in vitro* assay of colonization resistance. For vancomycin-treated patients, growth of *C. difficile* was suppressed in a majority of stool filtrates and suspensions during therapy and for 3-5 days after completion of therapy. By 6 days after discontinuation of CDI therapy, a majority of stool filtrates supported growth of *C. difficile*, consistent with the finding that inhibitory levels of vancomycin were no longer present. From 6 to 21 days after therapy, a majority of stool suspensions supported growth of *C. difficile*, whereas after 21 days most samples were inhibitory, suggesting that the components of the microbiota that provide colonization resistance had recovered sufficiently to inhibit growth.

For metronidazole-treated patients, growth of *C. difficile* was suppressed in stool filtrates and suspensions of samples with detectable metronidazole during treatment, but not in filtrates of samples with no detectable metronidazole. By 15 to 20 days after metronidazole therapy, a majority of stool suspensions inhibited *in vitro* growth of inoculated *C. difficile*.

### Microbiota signature of *C. difficile* colonization resistance

**Figure 4:**
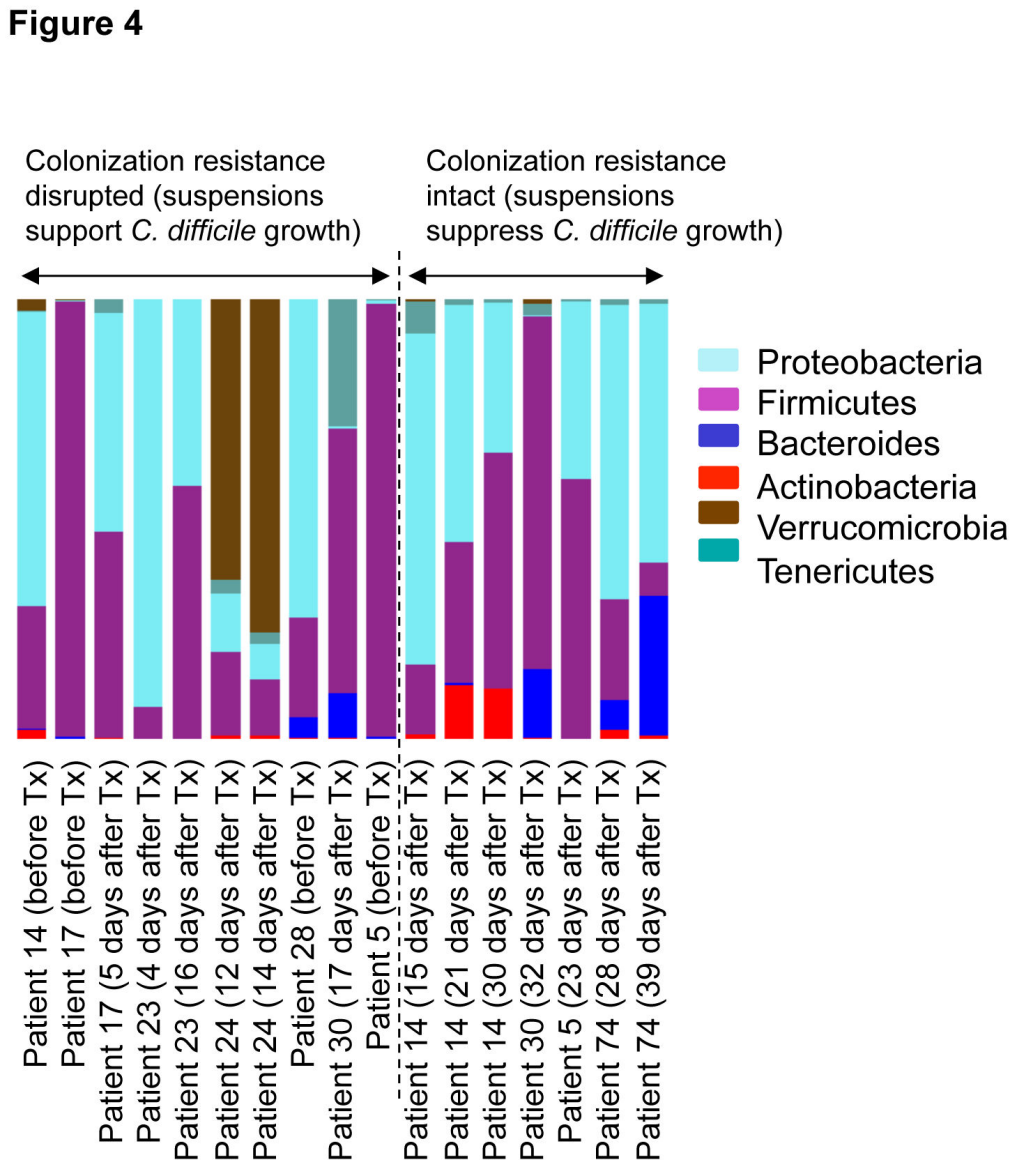
Relative abundance of bacterial phyla in stool of 8 patients treated with oral vancomycin. Stool specimens collected before or after completion of vancomycin therapy for Clostridium difficile infection (CDI), stratified by those with intact (i.e., *C. difficile* growth suppressed) or disrupted (i.e., *C. difficile* growth supported) colonization resistance based on the *in* *vitro* colonization assay. Micriobiota of each stool specimen was evaluated by deep 16S rRNA sequencing. Each column represents total phyla distribution of one sample. Columns are labelled with sample ID and timing of stool collection in comparison to vancomycin treatment.

**Figure 5:**
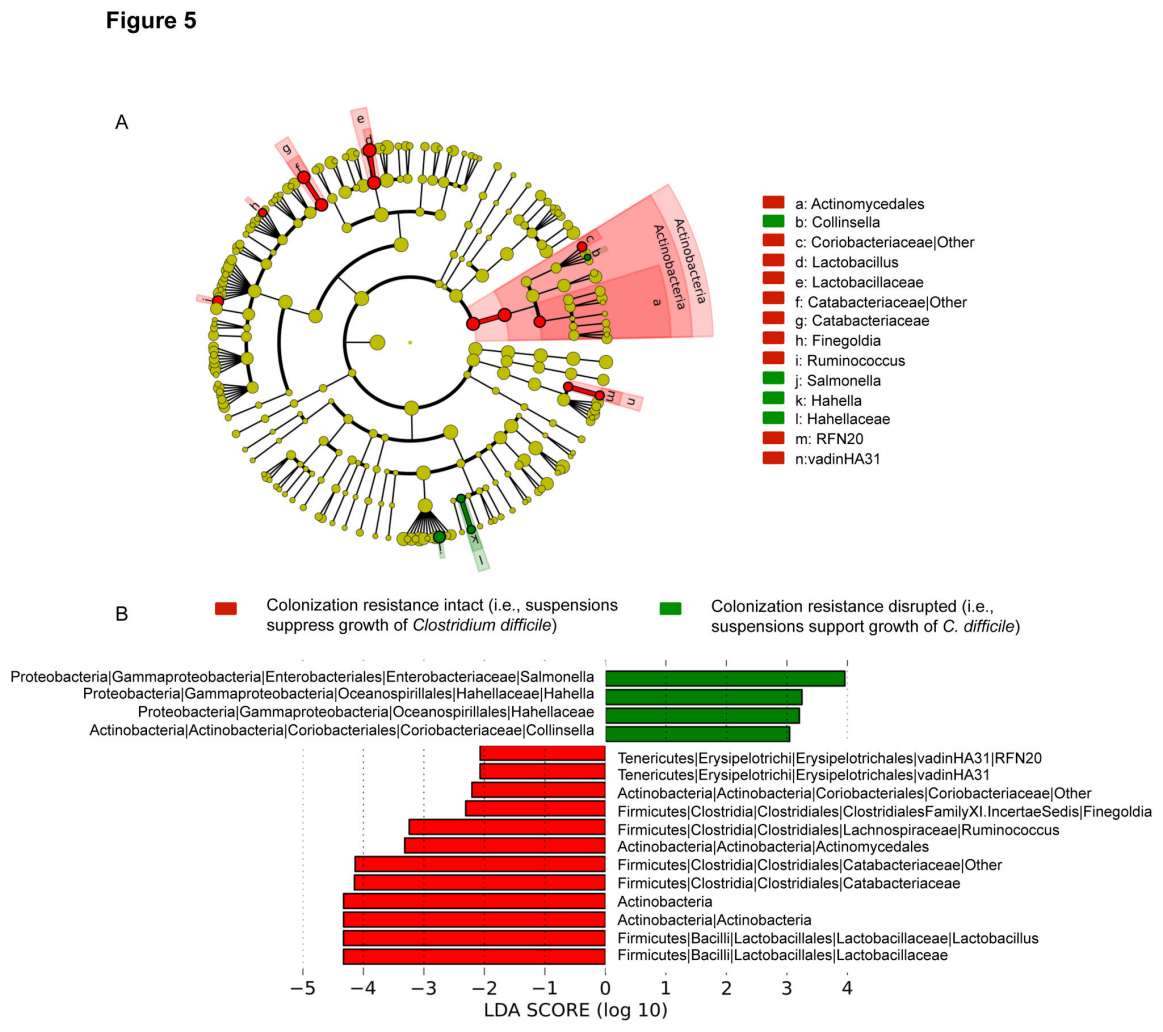
Microbiota signature of bacteria associated with intact versus disrupted colonization resistance to *Clostridium difficile*. (A) Phylogenetic tree display of the bacteria identified from stool samples of 8 vancomycin-treated patients with intact (i.e., *C. difficile* growth suppressed) or disrupted (i.e., *C. difficile* growth supported) colonization resistance based on the *in* *vitro* colonization assay. Clades of bacteria that significantly support or suppress *C. difficile* growth are highlighted in green or red, respectively. Circle diameter at each phylogenetic level is proportional to the corresponding taxon’s abundance. (B) Histogram of the Linear Discriminant Analysis (LDA) score of key bacteria abundance in suppressive and supportive groups arranged according to their effect size. Positive (green bars) and negative (red bars) LDA scores represent supportive and suppressive bacteria, respectively.

Microbiota relative abundance at different phylogenetic levels was studied in stool specimens that did or did not support growth of *C. difficile* based upon the *C. difficile in vitro* colonization assay; for this analysis, specimens collected during or within 5 days after CDI treatment were excluded because suppression of *C. difficile* may have been due to the CDI therapy. At the phylum level, *Actinobacteria* levels were higher (*P*=0.07) in specimens with intact colonization resistance (i.e., inhibition of *C. difficile* growth) (Figure 4). Bacterial biomarkers associated with intact or disrupted colonization resistance to *C. difficile* were detected using LEFSe approach [17]. Based on LEFSe analysis, three bacterial genera were associated with absence of colonization resistance to *C. difficile* (Figure 5). The two genera with the highest effect size (*Salmonella* and *Hahella*) belong to *Gammaproteobacteria* class of the phylum *Proteobacteria*, while the third genus (*Collinsella*) is related to the phylum *Actinobacteria*. In general, bacteria belonging to three phyla (*Actinobacteria*, *Firmicutes*, and *Tenericutes*) were associated with intact colonization resistance. The highest effect size was observed in the class *Actinobacteria* and the genus *Lactobacillus*. Other bacteria, arranged from highest to lowest effect size are unclassified genus of the *Catabacteriaceae* family, the order *Actinomycetales*, the genus *Ruminococcus*, the genus *Finegoldia*, an unclassified genus of the *Coriobacteriaceae* family, and an unidentified bacterium genus (RFN2) of *Tenericutes*. Also, the relative abundance of the genus *Bifidobacterium* was increased in resistant compared to sensitive samples (*P* = 0.055).

### Analysis of Stool Microbiota of Vancomycin-Treated Patients

**Figure 6:**
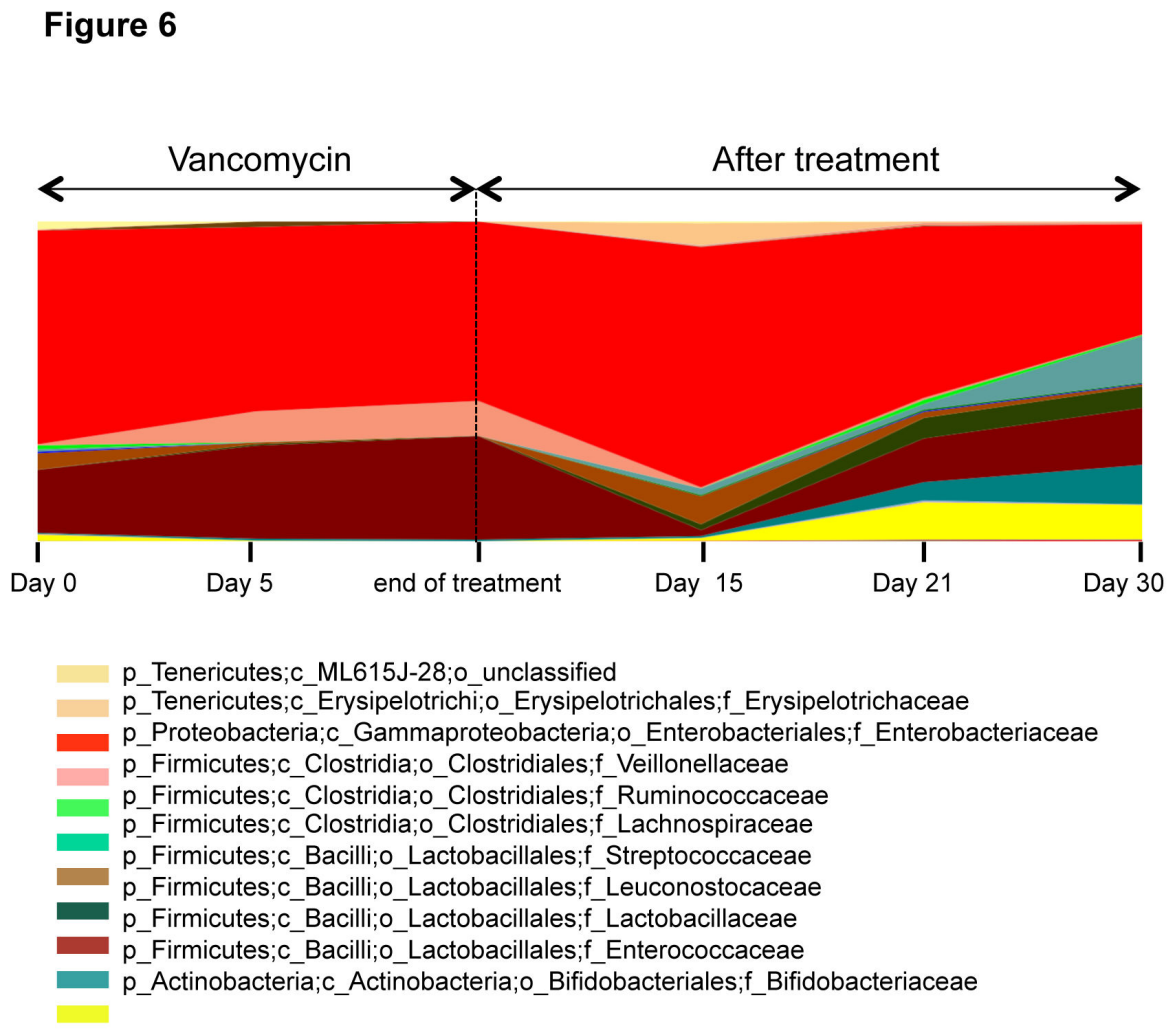
Surveillance of bacterial family relative abundance before, during, and after vancomycin treatment in one patient. Stool samples were collected before (Day 0), during, and after vancomycin treatment at the indicated time points. Changes in the abundance (abundance >0.05%) of major bacterial families are shown at each time point.

Figure 6 shows data for a single vancomycin-treated patient that illustrates the typical changes seen over time in the stool microbiota before, during, and after treatment. A total of ~ 14 million high quality reads were generated from all samples with an average reads count of ~ 0.5 million reads/sample. Before treatment, the microbiota was composed of 8 phyla, with *Proteobacteria* (67.1%), *Firmicutes* (27.7%), and *Actinobacteria* (2.2%) being most abundant. The remaining 3.1% was distributed among *Bacteroidetes*, *Cyanobacteria*, *Fusobacteria*, *Tenericutes*, and *Verrucomicrobia* (data not shown). During vancomycin treatment, there was a general decrease in different families of the *Firmicutes* phylum including *Streptococcaceae*, *Lachnospiraceae*, *Leuconostocaceae*, and *Ruminococcaceae*. In addition to *Firmicutes* phylum, a marked decrease was also observed in the *Proteobacteria*, *Actinobacteria* and *Tenericutes* phyla represented by *Enterobacteriaceae*, *Bifidobacteriaceae*, and *Erysipelotrichaceae* families; respectively. This decrease in relative abundance reached its lowest level at the end of treatment period.

Following treatment, two main trends were observed in families with reduced abundance during treatment. First, there was a sudden increase in bacterial relative abundance 15-20 days following treatment. Second, the bacteria relative abundance dropped to a level lower than the pre-treatment state. This was seen with *Streptococcaceae*, *Ruminococcaceae*, and *Enterobacteriaceae*. The second trend was characterized by an increase in relative abundance following treatment over a 30 day period, in which it exceeded the original pre-treatment state. This was observed with *Bifidobacteriaceae*, *Leuconostocaceae*, *Lachnospiraceae*, and *Erysipelotrichaceae*.

## Discussion

In an observational study of CDI patients, we found that vancomycin-treated patients had high levels of vancomycin in stool during therapy that typically decreased to undetectable levels by 6 to 10 days after discontinuation of therapy. In contrast, metronidazole-treated patients had low levels of drug present during treatment that decreased to undetectable levels by the end of CDI therapy. For both drugs, a significant proportion of fresh stool suspensions from the period shortly after completion of therapy (~6 to 21 days after completion of vancomycin and from end of therapy to 14 days post-treatment for metronidazole) did not inhibit growth of inoculated *C. difficile*, suggesting that the microbiota that provide colonization resistance against *C. difficile* had not yet recovered. Moreover, vancomycin-treated patients frequently developed recurrent colonization with *C. difficile* 10 to 14 days after completion of therapy, whereas metronidazole-treated patients did not. These results suggest that there is a vulnerable period for re-establishment of *C. difficile* colonization after oral vancomycin or metronidazole therapy that extends from the time when drug levels are reduced to subinhibitory concentrations to the time when the microbiota are sufficiently recovered to inhibit growth of *C. difficile*.

Previous studies have demonstrated that oral vancomycin may cause marked and prolonged suppression of the indigenous microbiota of the colon, including *Bacteroides* spp. [4,5]. Our data confirm those observations. Moreover, our findings demonstrate that oral metronidazole therapy may also be associated with prolonged disruption of the microbiota. Although previous studies have demonstrated that disturbances of the microbiota may be prolonged, they have not included a functional assessment of the ability of the microbiota to inhibit colonization by *C. difficile*. Our findings from the *in vitro* assay of colonization resistance suggest that functional recovery of colonization resistance may precede full recovery of the diversity of the microbiota. Our *in vitro* data are supported by the finding that *C. difficile* was frequently acquired at 10 to 14 days but not later after discontinuation of vancomycin therapy. Similarly, Johnson et al. [18] found that vancomycin, but not metronidazole, treatment suppressed *C. difficile* in stool of asymptomatic carriers, but that 8 of 9 carriers again began shedding spores 20 +8 days after completing treatment.

Based upon deep 16SrRNA sequencing analysis, the presence of bacteria belonging to three phyla (*Actinobacteria*, *Firmicutes*, and *Tenericutes*) was associated with intact colonization resistance. The class *Actinobacteria* and the genus *Lactobacillus* had the strongest association with intact colonization resistance to *C. difficile. Actinobacteria* is a very large class that encompasses about 300 different bacterial genera, including *Bifidobacteriaceae*. We found that the family *Bifidobacteriaceae* regained colonization in the gut following vancomycin treatment at a level higher than the pretreatment state. Of particular interest, the relative abundance of the genus *Bifidobacterium* was increased in samples with intact colonization resistance. Although the efficacy of probiotics for prevention of CDI is controversial, some studies suggest that *Lactobacillus* and *Bifidobacterium*, when used as probiotics, may be associated with reduced CDI [19,20].

*Ruminococcus* spp. was also associated with intact colonization resistance in this study. *Ruminococcus* strains are known to produce the lantibiotic Ruminococcin A (RumA) in the presence of trypsin, which inhibit the in vitro growth of various pathogenic bacteria including *C. difficile* and *C. perfringens* [21]. LEFSe has highlighted the family of *Catabacteriaceae* within the group of colonization resistant bacteria. *Catabacteriaceae* is a recently identified family which comprises one descendant, *Catabacter hongkongensis* [22,23]. The role of *C. hongkongensis* in the human gut and its microbial community is unknown. Similarly, the role of *Finegoldia* and *Coriobacteriaceae* in *C. difficile* colonization resistance requires further investigation. Finally, although the unidentified bacterium genus (RFN2) of *Tenericutes* was identified among the bacteria associated with colonization resistance, their increased abundance following vancomycin treatment may also be attributed to their natural resistance to vancomycin [24,25]. *Salmonella*, *Hahella* and *Collinsella* were associated with disrupted colonization resistance.

Our findings have important implications for development of strategies to prevent recurrences of CDI. For example, nontoxigenic *C. difficile* strains are in Phase 2 trials for secondary prevention of CDI [6,26]. Knowledge of the vulnerable period for re-establishment of *C. difficile* colonization may be useful for optimization of the timing of dosing of this agent. In addition, this knowledge could be used to optimize the timing of other biotherapeutic agents being trialed for prevention of recurrences. Other infection control measures (e.g., environmental disinfection or bathing to reduce the burden of spores on skin) may also be most beneficial if targeted to the vulnerable period.

Our study has several limitations. The study population included mostly elderly men with multiple medical problems. Additional assessments of the impact of CDI therapies in other populations are needed. Because the study was observational, it is possible that the longer period of disruption of colonization resistance in the vancomycin versus metronidazole-treated patients was attributable to the fact that vancomycin-treated subjects were more likely to have severe CDI, rather than to differences in the 2 drugs. However, the longer period of disruption of colonization resistance by vancomycin is consistent with the higher and more prolonged levels of drug achieved in the intestinal tract. The in vitro assay of colonization resistance has been validated with in vivo findings in hamsters [13] and mice (authors’ unpublished data), but further validation of this method is required in humans.

In summary, we demonstrated that that there is a vulnerable period for re-establishment of *C. difficile* colonization after CDI treatment that begins when levels of vancomycin or metronidazole reach subinhibitory concentrations and extends for about 3 weeks in most patients. These findings have important implications for development of effective interventions to prevent recurrences of CDI.
